# Photo- and Sono-Active Food Colorants Inactivating Bacteria

**DOI:** 10.3390/ijms242015126

**Published:** 2023-10-12

**Authors:** Efrat Hochma, Iryna Hovor, Faina Nakonechny, Marina Nisnevitch

**Affiliations:** Department of Chemical Engineering, Ariel University, Ariel 4070000, Israel; efratho@ariel.ac.il (E.H.); irynah@ariel.ac.il (I.H.); fainan@ariel.ac.il (F.N.)

**Keywords:** food colorants, photosensitizers, sonosensitizers, *S. aureus*, *E. coli*, Erythrosine B (E127), Rhein, E129 (Allura Red AC), E124 (Ponceau 4R), E122 (Azorubine), E150a (caramel and vanillin)

## Abstract

Food colorants are commonly used as excipients in pharmaceutical and nutraceutical fields, but they have a wide range of other potential applications, for instance, as cytotoxic drugs or mediators of physical antimicrobial treatments. The photodynamic antibacterial activity of several edible food colorants is reported here, including E127, E129, E124, E122, E133, and E150a, alongside Rhein, a natural lipophilic antibacterial and anticancer compound found in medicinal plants. Minimal inhibitory concentration (MIC) values for *S. aureus* and *E. coli* showed that E127 and Rhein were effective against both bacteria, while other colorants exhibited low activity against *E. coli*. In some cases, dark pre-incubation of the colorants with Gram-positive *S. aureus* increased their photodynamic activity. Adding Rhein to E127 increased the photodynamic activity of the latter in a supportive mode. Optional sensing mechanism pathways of combined E127/Rhein action were suggested. The antibacterial activity of the studied colorants can be ranged as follows: E127/Rhein >> E127 >> E150a > E122 > E124 >> E129 ≈ E133. E127 was also found to exhibit photodynamic properties. Short ultrasonic treatment before illumination caused intensification of E127 photodynamic activity against *E. coli* when applied alone and especially in combination with Rhein. Food colorants exhibiting photo- and sonodynamic properties may have good potential in food preservation.

## 1. Introduction

In recent years, public interest in food additives has increased greatly [1]. While colorants are commonly used as excipients in pharmaceutical and nutraceutical applications, they also have a wide range of potential applications in the medical, environmental, and food industries as cytotoxic drugs and mediators of physical antimicrobial treatments [2,3].

In the food industry, the efficient reduction of initial microbial loads is especially crucial. With the advancement of technology, the demand has increased significantly for healthy “green” foods without chemical preservatives that retain nutritional and sensory attributes, as well as microbial safety. In addition, freshness, naturalness, and minimal processing are also appreciated. Therefore, nonthermal sanitizing methods have been studied as a superior strategy to eliminate the heat destruction of a food’s organoleptic properties [3,4,5,6].

Photodynamic antimicrobial treatment (PACT) has been proposed and used as a method of treating bacteria, fungi, or viruses found in the body, food, or environment. PACT uses a dye molecule called a photosensitizer (PS) that can generate cytotoxic reactive oxygen species (ROS) when exposed to visible light. The photophysical and photochemical cascade reactions in the presence of ROS result in the destruction of bacteria [7,8,9,10,11,12,13,14].

To overcome the PACT light-penetration handicap, a new strategy, sonodynamic treatment (SDT), was suggested [8,15,16]. Ultrasound (US) is a broad-scale method for the activation of sensitizers, performing the deep-phase penetration lacking in PACT. Moreover, US may provide a uniform dispersion of the sonosensitizer in the treated medium and contribute to the destruction of the cell membrane of bacteria.

Sono-photodynamic treatment is a strategy combining the advantages of PACT and SDT [8,17]. It is hypothesized that the sono-photosensitizer can be excited by both light and US, thus increasing the cytotoxic effect [8]. Using light in combination with US offers a promising alternative to overcome the individual limitations of PACT and SDT, since US exhibits excellent permeability, while light stimulates the sensitizer. The enhanced sensitizer performance results in improved ROS generation.

Some food colorants, such as Erythrosine B (E127), may exhibit photo- and/or sonodynamic properties. E127 is a red xanthene dye that absorbs maximum light at a wavelength of 525 nm (at pH 7). It is used in red-colored foods and potentially can mediate physical disinfection treatments, including photodynamic treatment. Erythrosine B is the only xanthene dye approved in the United States and the EU, and it is widely used orally in pharmaceutical doses [18]. Its positive photoactive response has been widely reviewed. However, it is associated with higher health concerns than other red synthetic dyes. The E127 dye concentration should range from 0.0005% to 0.001% in liquid preparations. Therefore, its regulatory limit is an essential consideration for its use [2,19,20,21,22,23,24]. Another yellowish dye of natural edible plant origin, Rhein, was previously reported to have antimicrobial and anticancer activity [25,26,27], but it was not studied for its photo- or sonodynamic activity.

The purpose of the work was to find out if edible colorants widely used in the food industry can also serve as food disinfectants when excited by visible light or ultrasound. This possibility may be useful for food preservation improving food safety and quality. The present work reports on the antibacterial properties of a number of food colorants and of Rhein under photo- and sonodynamic treatments.

## 2. Results and Discussion

In this work, we studied and analyzed the photodynamic activity against Gram-positive *S. aureus* and Gram-negative *E. coli* exhibited by commonly used edible food colorants: E127, E129, E124, E122, E133, and E150a. Additionally, Rhein, a yellowish dye of edible plant origin with antimicrobial [25,27,28,29] and anticancer [26] activity, was investigated for its photodynamic and sonodynamic properties when applied alone or in combination with E127 to inhibit Gram-negative *E. coli*.

### 2.1. Spectral Characterization of the Colorants

The full names, structures, and wavelength values at maximal absorption (λ_max_) for the studied colorants are presented in Table 1.

**Figure 1:**
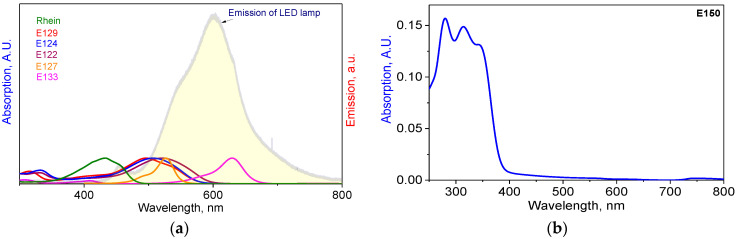
The spectral overlap between absorption spectra of the colorants and the emission spectrum of the LED lamp (**a**) and the absorption spectrum of E150a (**b**).

Absorption spectra of all the studied colorants were measured and compared to the emission spectrum of the white LED lamp used in this study (Figure 1). The absorption spectrum of E150a is shown separately in Figure 1b. E150a is composed of two main components: uncolored vanillin and colored caramel [30]. Only the latter can exhibit photodynamic activity since caramel is a complex mixture of compounds with an undefined structure, including caramel olefins and alkynes (Table 1), having conjugated double and triple bonds and causing light absorption between 720 and 800 nm (Figure 1b). Vanillin absorbs in the UV region only and cannot be excited by an LED lamp having an emission spectrum in a visible range only (Figure 1a).

It can be seen from Figure 1 that in all the cases, the absorption spectra have a full (E133) or at least partial (E129, E124, E122, E127 and Rhein) overlap with the emission spectrum of the white LED lamp; therefore, this lamp can be applied in photodynamic studies. It should be noted that all the colorants used here exhibit strong light absorption in the UV region, especially E150a, due to the presence of vanillin. However, since the LED lamp emits visual light only, we did not relate to the spectra of the colorants in the UV region.

### 2.2. Determination of MIC Values of Food Colorants against S. aureus and E. coli

**Figure 2:**
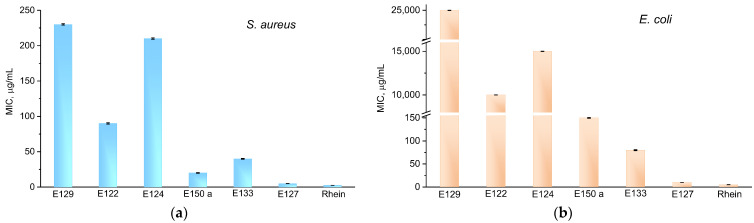
MIC concentrations of food colorants after 30 min white light illumination on *S. aureus* (**a**) and *E. coli* (**b**).

To determine whether the food colorants exhibited antibacterial properties, their MIC values were determined for Gram-positive *S. aureus* and Gram-negative *E. coli* bacteria under continuous white LED illumination (Figure 2). The results showed that in all the cases, the growth of *S. aureus* was suppressed at concentrations between 2.5 and 230 μg/mL (Figure 2a). Another picture was seen in the case of *E. coli*. Colorants E127, E133, E150, and Rhein were effective against *E. coli*, but E122, E124, and E129 showed very low activity, with MIC values ranging between 10 and 25 mg/mL—about 100 times higher than in the case of *S. aureus* (Figure 2b). E127 and Rhein showed the highest inhibitory effect against *E. coli*. The suppression of bacterial cells occurred at concentrations of 10 μg/mL for E127 and 5 μg/mL for Rhein—only two times higher than in the case of *S. aureus*.

### 2.3. Photodynamic Activity of Food Colorants against S. aureus and E. coli

**Figure 3:**
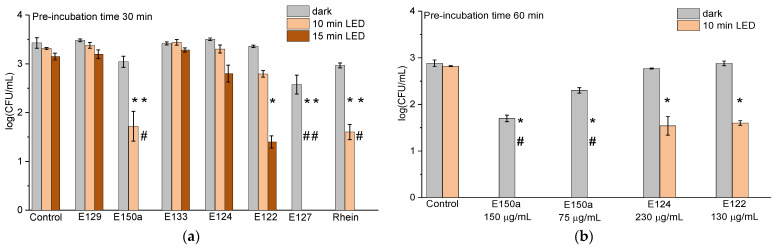
The effect of studied colorants on suspensions of 10^3^ CFU/mL *S. aureus* mixed with the colorants at final concentrations of (**a**) 290 µg/mL (E129), 300 µg/mL (vanillin in E150a), 70 µg/mL (E133), 230 µg/mL (E124), 130 µg/mL (E122), 80 µg/mL (E127), and 4.5 µg/mL (Rhein) after 30 min dark pre-incubation; (**b**) 75 and 150 µg/mL (vanillin in E150a); 230 µg/mL (E124); and 130 µg/mL (E122) after 60 min dark pre-incubation and upon further exposure to white LED light. * indicates a significant difference (*p* < 0.05) between the sample groups and the control. # indicates the total inhibition of bacterial cells. Error bars represent the STDEV.

**Figure 4:**
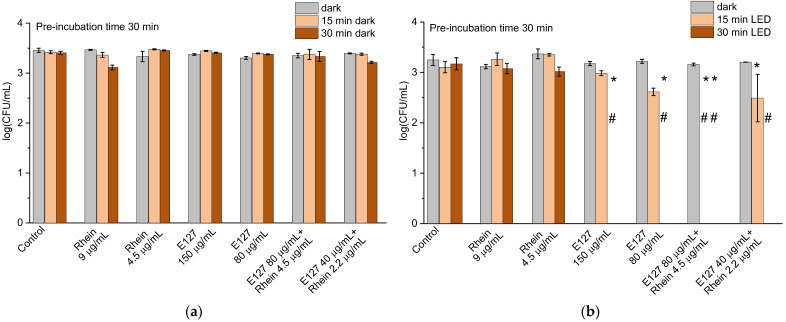
The effect of E127, Rhein, and E127/Rhein combination on *E. coli* at 10^3^ CFU/mL, with 15 min and 30 min dark incubation (**a**) and under white LED illumination for 15 min and 30 min (**b**). * indicates a significant difference (*p* < 0.05) between the sample groups and the control. # indicates the total inhibition of bacterial cells. Error bars represent the STDEV. Dark pre-incubation was 30 min.

To find out whether the antibacterial activity of food colorants and Rhein can be attributed to a photodynamic effect, the viability of *S. aureus* and *E. coli* was studied under white LED illumination and in the dark. For this purpose, the colorant–bacteria mixtures were pre-incubated in the dark for 30 min and then either illuminated or kept in the dark as a control. The results of these experiments are presented in Figure 3 and Figure 4. As can be seen there, the colorants E129 and E133 showed no photodynamic activity against *S. aureus* (Figure 3a). The results obtained in their presence did not differ from the controls (*p*-values were 0.10 and 0.12 for E129 and E133, respectively). For this reason, these colorants were not studied further.

E124 and E122 showed some signs of photosensitization potential. Light irradiation of bacteria with these colorants for 15 min reduced the bacterial concentration by 0.6 log_10_ (E124) and 2 log_10_ (E122). It should be noted that for E124, this difference was not statistically proven (*p*-value = 0.27). Rhein showed high antibacterial activity. After 10 min of irradiation, the concentration of live cells decreased by 1.5 log_10_, and after 15 min, the bacteria were completely killed. The E150a colorant, containing vanillin, killed *S. aureus* very effectively (Figure 3a); a decrease in the *S. aureus* concentration of one order of magnitude was registered after 10 min of illumination, and total cell elimination was achieved after 15 min.

Since in many cases the efficacy of photodynamic treatment depends on the penetration of PSs into bacterial cells [31], the pre-incubation time was increased to 60 min to enhance the possibility of PS penetration into *S. aureus* cells. Prolonging the dark pre-incubation time affected the photodynamic activity of E122 and E124 (Figure 3b). In both cases, the colorants caused a 1.5 log_10_ drop in *S. aureus* concentration after 10 min illumination.

Since E150a showed a sign of dark activity (Figure 3a), this colorant was also examined under prolonged dark pre-incubation, and its concentration was varied. Dark 60 min pre-incubation of *S. aureus* with E150a at a concentration of 300 µg/mL led to a complete inhibition of cells, whereas at the concentration of 75 µg/mL, dark toxicity caused a 0.5 log_10_ reduction in cell concentration, and at 150 µg/mL–1.2 log_10_. In this case, the *p*-values were 0.04 and 0.05, respectively, compared to the controls. After the subsequent 10 min illumination, all the bacteria were inactivated (Figure 3b). We assume that the exhibited antibacterial properties of E150a were preponderately due to the dark toxicity of vanillin, which is known for its antibacterial properties [32]. Nevertheless, E150a showed photodynamic activity as well; this cannot be caused by vanillin since the latter absorbs light in a UV region only, and illumination was performed by visible light only (Figure 1). Additionally, the color of E150a is conditioned by caramelized sugars (Table 1), having multiple unsaturated bonds. Although caramel does not possess any unambiguous structure, caramel olefins and alkynes could be responsible for E150a photodynamic activity.

E127 was the most active agent, totally destroying *S. aureus* bacteria cells already after 10 min of illumination. E127 is a well-known PS [33,34], and its photodynamic properties are well explained by multiple conjugated double bonds and a presence of halogen atoms in its structure (Table 1). E127 has a high quantum yield of ROS production of 0.62 [35].

**Figure A1:**
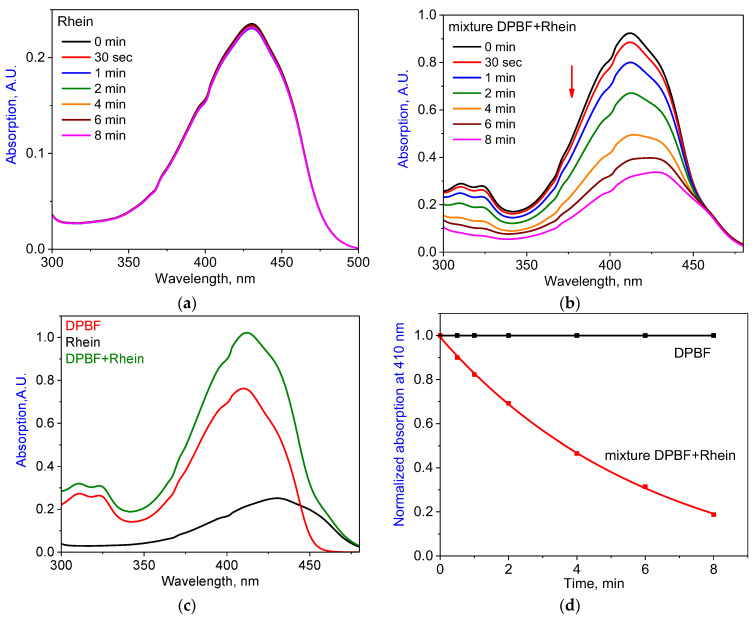
Testing of ROS production by Rhein under illumination. Absorption spectra of Rhein alone (**a**), DPBF in the presence of Rhein (**b**), and DPBF (**c**) alone under illumination. (**d**) Normalized absorption of DPBF at 410 nm alone and in the presence of Rhein.

The photodynamic activity of Rhein is not described in the literature; therefore, the testing for ROS production was performed using DPBF as a fluorescent probe for the detection of ROS [36]. In this experiment, we simultaneously illuminated one cuvette with Rhein solution alone, a second with Rhein and DPBF, and the third with DPBF alone. The results, presented in Figure A1 (Appendix A), showed that the spectrum of Rhein did not change upon illumination (Figure A1a), whereas absorption of the Rhein–DPBF mixture decreased in time (Figure A1b).

The problem was that the absorption spectrum of DPBF (λ_max_ 410 nm), as with many other indicators for ROS [36,37], overlapped with the Rhein spectrum (λ_max_ 433 nm) (Figure A1c), making it very challenging to distinguish changes in absorption of DPBF vs. Rhein. To overcome this difficulty, the absorption of Rhein at 410 nm was subtracted from the spectrum of Rhein–DPBF. Such an approach was applied previously when studying ROS production by aloe emodin [38]. The changes in DPBF absorption at 410 nm in the presence of Rhein, relative to absorption of DPBF alone, are presented in Figure A1d. The latter graph shows that Rhein definitely produced ROS under illumination and can be considered a PS. However, it seemed inappropriate to calculate the quantum yield of ROS production using these data, since the experimental error was undoubtedly high.

Further, we studied the possibility of applying food colorants against Gram-negative *E. coli.* It is known that Gram-negative bacteria are more persistent to photodynamic treatment [8,17]. This feature may be attributed to their cell envelope structures, which are different from Gram-positive cells. Gram-negative bacteria possess less permeable cell walls due to a double lipid bilayer sandwiching the thin, porous peptidoglycan layer, in addition to an outer lipopolysaccharide layer with numerous negatively charged molecules. All of these reduce permeability and the attachment of neutral PSs or repel anionic ones [8,10,16]. For these reasons, we chose to investigate only those colorants which were the most active against *S. aureus*, namely, E127 and Rhein. According to previously published data, the activity of some antibacterial agents could be enhanced by adding Rhein [27,29]; therefore, we studied not only individual compounds but their mixture as well. This combination has not been previously applied against bacteria. In this experiment, we varied colorant concentrations and illumination time. Parallel experiments were performed in the dark and under illumination. The results are shown in Figure 4.

The colorants did not reveal any significant dark activity in any of the studied concentrations (Figure 4a). However, under illumination, at the concentrations of 80 and 150 μg/mL, E127 completely destroyed the *E. coli* cells after half an hour, whereas after 15 min, the cells were inactivated only partially. This result showed that E127 can be considered a strong photodynamic antibacterial agent. Rhein alone at 4.5 µg/mL and 9 µg/mL concentrations had almost no effect on *E. coli* (Figure 4b). A different picture was obtained when E127 was combined with Rhein. In the mixture of 80 µg/mL E127 and 4.5 µg/mL Rhein, the cells were totally inactivated already after 15 min (Figure 4b), whereas during an equivalent period, the individual components hardly affected the cells. Even at half concentrations of both components, the decimation of *E. coli* was observed after 30 min illumination. We assume that these data imply a supportive mode of interaction between E127 and Rhein.

Previously, it was reported that Rhein can participate in synergistic actions with antibiotics [29]. However, no mechanism for explaining this phenomenon was suggested.

Undoubtedly, the ability of each component of ROS production causes the amplification of free radicals in the system, leading to effective bacterial kill, but this phenomenon does not explain the supportive action of E127 and Rhein.

Possible explanations of the supportive action of E127 and Rhein under illumination may be FRET (Förster resonance energy transfer) or PET (photoinduced electron transfer) interaction between the molecules of colorants. E127 can act as an electron acceptor [39], and Rhein, having electron-donating OH groups, could perform electron transfer. Since E127 is a very hydrophilic compound and exhibits a low interaction with biological membranes [35], the interaction between the colorants is more likely to occur in the solution or in the cytoplasm, which is composed mainly of water, rather than in hydrophobic parts of cells. For this reason, the fluorescent studies necessary to evaluate the interaction mechanism were carried out in saline.

**Figure 5:**
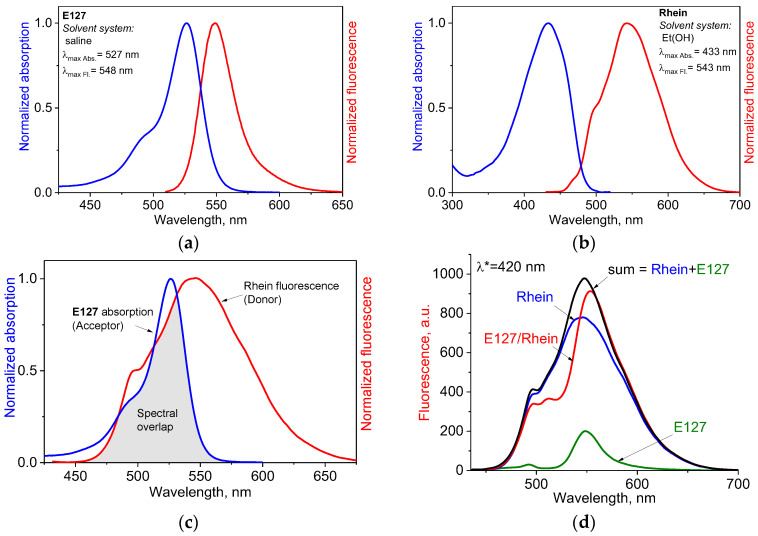
Absorption (blue) and emission (red) spectra of E127 (**a**) and Rhein (**b**) in saline; the spectral overlap between the fluorescence spectrum of Rhein (red) and the absorption spectrum of E127 (blue) in saline (**c**); fluorescence spectra of E127 (green), Rhein (blue), and E127/Rhein mixture (red) in saline at excitation wavelength λ* = 420 nm and a calculated mathematical sum of E127 and Rhein (black) (**d**). Concentrations of E127 and Rhein were 30 µg/mL and 16 µg/mL, respectively.

Figure 5a,b presents the absorption and emission spectra of E127 and Rhein, respectively. The fluorescent spectrum of Rhein (λ_max Fl_ = 543 nm) substantially overlaps the absorption spectrum of E127 (λ_max Abs_ = 527 nm) (Figure 5c). This observation indicates that these colorants can interact as a donor–acceptor pair.

Quantum yields of fluorescence (Φ_F_) measured for both colorants showed very low values; for E127, the quantum yield was Φ_F_ = 0.02, which is consistent with the literature data [35,40], and for Rhein, it was Φ_F_ = 0.01.

In the case of a FRET interaction of two colorants, the donor emission should decrease, and the acceptor emission is expected to increase, due to the transfer of energy from the excited donor to the acceptor [41]. Also, in the case of FRET, the mathematical sum of the donor and the acceptor spectra should not coincide with the experimental spectrum of their mixture. When PET occurs, the fluorescence of the acceptor is expected to be quenched since the acceptor is converted into a radical anion by photo-induced electron transfer [42].

To understand if FRET or PET interactions may occur between E127 and Rhein, we examined the fluorescence spectra of both components alone and in a mixture in saline, using excitation at the wavelength 420 nm. This wavelength was chosen to provide excitation of a donor without causing the excitation of the acceptor (Figure 5a,b).

The results of the experiment are presented in Figure 5d. As can be seen, the emission spectrum of the E127/Rhein mixture (red line) differed from the calculated mathematical sum of both components’ spectra (black line). The difference between experimental and theoretical spectra can be explained as decreases in Rhein emission and increases in E127 emission by FRET interaction. However, the impact of the FRET interaction on the overall effect cannot be very substantial due to a low fluorescent quantum yield of Rhein in saline (Ф_F_ = 0.01).

In the case of PET, the fluorescence of E127 as an acceptor is expected to be quenched [43,44,45,46], and the emission spectrum of the mixture should not exceed the spectrum of the donor. However, the spectrum of the mixture showed unquenched E127 fluorescence (Figure 5d). For this reason, we concluded that the probability of PET is very low.

Another explanation for the increased activity of the E127–Rhein mixture may include the biochemical activity of Rhein, which supports the photodynamic action of E127. Suggested biochemical paths may include the blocking of NADH dehydrogenase-2 activity [47], an increase in the cell permeability, causing leakage of cell contents [48,49], or increasing the resistance of bacteria to adverse factors at the gene level [50,51].

It also seems possible that Rhein reduces the resistance of bacteria to the ROS generated by E127, thus increasing the overall photodynamic effect.

However, the exact mechanism of the supportive action between E127 and Rhein is unclear.

### 2.4. Inactivation of E. coli by Sono- and Photodynamic Treatment in Series Mediated by E127, Rhein, and E127/Rhein Combination

In our previous works, we reported on the antibacterial sonodynamic activity of PSs, particularly Rose Bengal [52,53], which structurally is very close to E127. Here, we examined the sonodynamic activity of E127 and investigated whether ultrasonic treatment might improve or replace the photodynamic inactivation of *E. coli* by E127, Rhein, and their combination. In the control experiments, the *E. coli* cells were treated by ultrasound only.

**Figure A2:**
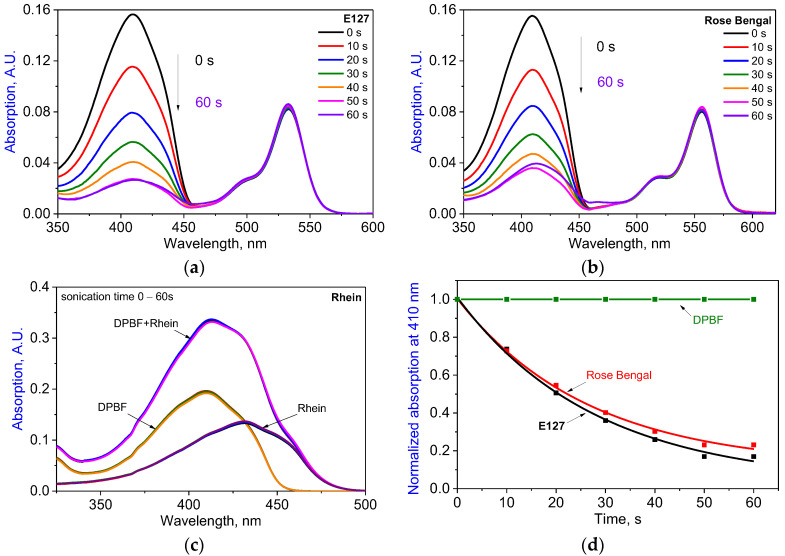
Testing ROS production by E127 and Rhein under sonication. Absorption spectra of DPBF in the presence of E127 (**a**), RB (**b**), and Rhein (**c**) under sonication. (**d**) Normalized absorption of DPBF at 410 nm alone and in the presence of E127 and RB.

First, an experiment was conducted to determine if ultrasonic treatment caused ROS production by E127 and Rhein. E127, Rhein, DPBF alone, and DPBF mixed with E127 and Rhein were subjected to sonication while monitoring their spectra in real time. In addition, as a positive control to ROS production under sonication, we used Rose Bengal, which is well known for its sonodynamic properties [54]. The results are presented in Figure A2 (Appendix A). As can be seen, the absorption of DPBF decayed rapidly in the mixture with E127, thus confirming the sonodynamic properties of the latter (Figure A2a). A similar picture was seen in the case of Rose Bengal (Figure A2b), indicating the closely similar sonodynamic properties of these compounds. The decay curves of DPBF in the presence of E127 and Rose Bengal are shown in Figure A2c. However, the behavior of DPBF in the presence of Rhein was different: the absorbance of DPBF and Rhein alone and of their mixture did not change with time (Figure A2d). The production of ROS under sonication of E127 was registered already after a very short treatment, less than a minute. For this reason, in further experiments, the chosen time for ultrasonic treatment of bacterial cells was very short, 10 or 30 s.

**Figure 6:**
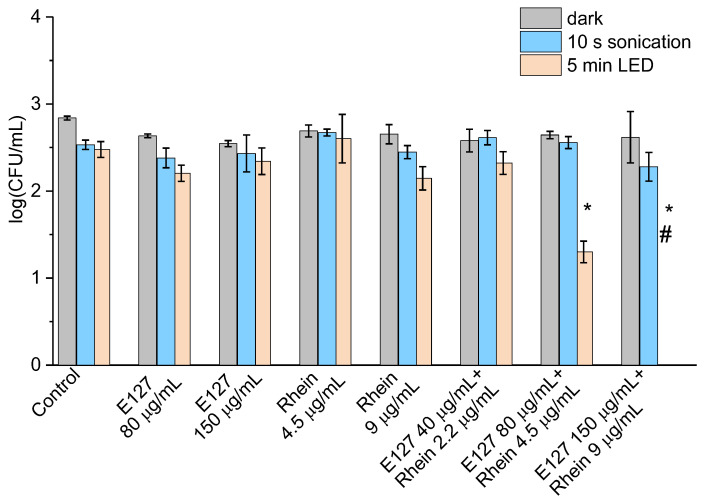
The effect of E127, Rhein, and E127/Rhein combination in the inactivation of *E. coli* cells at concentrations of 10^3^ CFU/mL, upon sono- and photodynamic treatment in series. * indicates a significant difference (*p* < 0.05) between the sample groups and the control. # indicates the total inhibition of bacterial cells. Error bars represent the STDEV.

As we showed previously, the complete elimination of *E. coli* was achieved with the combination of E127 (80 µg/mL) and Rhein (4.5 µg/mL) in 15 min of illumination, when the mixture was pre-incubated for 30 min in the dark (see Figure 4b). Here, we used short sonication and short irradiation time periods to reduce the total processing time and to find out whether this approach can be more effective in cell elimination than prolonged irradiation. In all the cases, the mixture of colorants with bacteria was not subjected to dark pre-incubation. Figure 6 shows that neither component separately nor their mixture reduced *E. coli* viability after 10 s sonication. In contrast, 5 min illumination following the sonication reduced cell concentration by more than 1 log_10_ when treated by the mixture of E127 (80 µg/mL) and Rhein (4.5 µg/mL) and caused cell elimination when treated by the mixture of E127 (150 µg/mL) and Rhein (9 µg/mL). It should be noted that none of the components alone caused any significant reduction in cell concentration under these treatment conditions (Figure 6).

**Figure 7:**
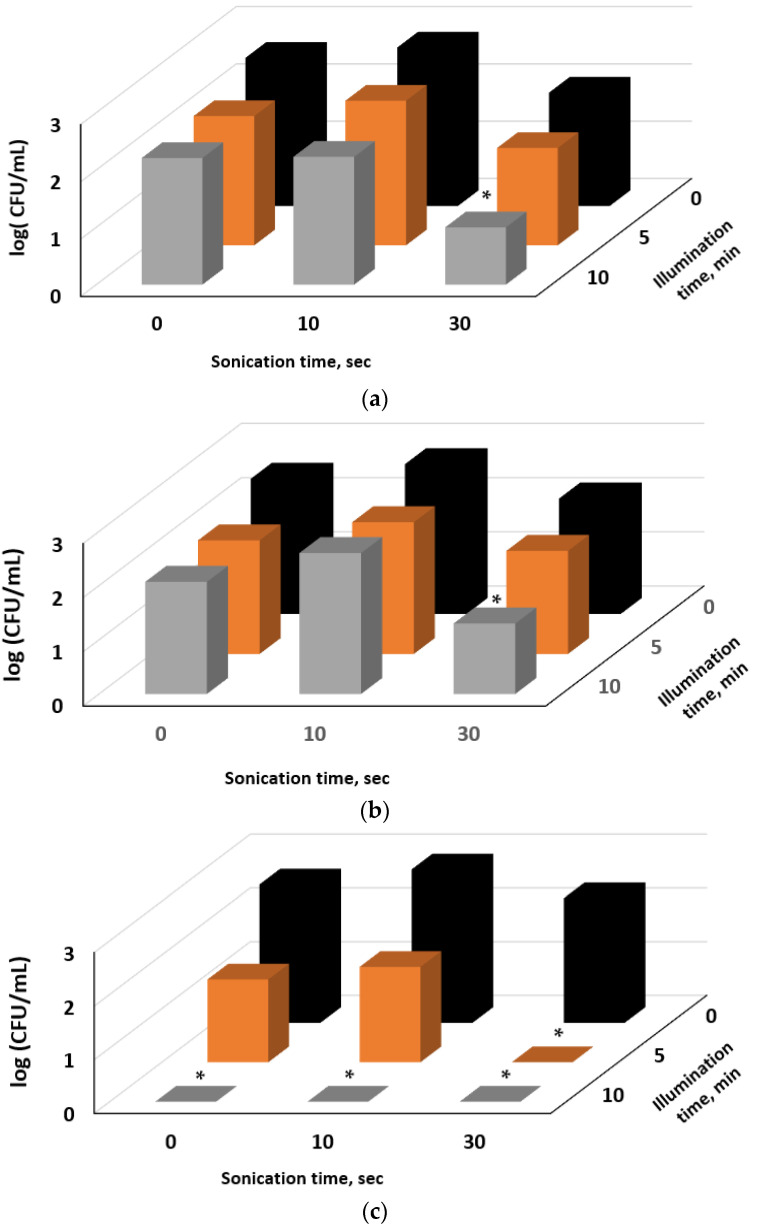
The effect of E127(**a**), Rhein (**b**), and E127/Rhein (**c**) combination on the inactivation of *E. coli* cells by ultrasonic pre-treatment followed by illumination. Concentration of bacterial cells 10^3^ CFU/mL, E127—150 µg/mL (**a**), Rhein—9 µg/mL (**b**), and combination of E127 80 µg/mL with 4.5 µg/mL Rhein (**c**). * indicates a significant difference (*p* < 0.05) between the sample groups and the control.

In the next stage, we investigated the effect of illumination and sonication time on the inhibition of bacteria by E127 and Rhein separately (Figure 7a,b, respectively) and by their mixture, when each component was applied at half the original concentrations (Figure 7c). The mixtures were not pre-incubated in the dark as in the previous experiment. As one can see from Figure 7a,b, each of the components separately caused only a moderate reduction in bacterial cells upon sonication for 30 s followed by illumination for 10 min. However, cell treatment by the component mixture at half concentration of each component showed very promising results (Figure 7c). The total elimination of cells was registered already after 30 s sonication followed by 5 min illumination, while 10 min illumination gave the same result with or without sonication. The sonodynamic activity of E127 decreased bacterial viability and facilitated *E. coli* cell death under the combined photodynamic action of E127 and Rhein. According to the obtained results, even short ultrasonic pre-treatment may replace the dark pre-incubation in which PSs attach to or penetrate cell envelopes. The results of this experiment once more confirmed our previous conclusion regarding the supportive action of E127 and Rhein.

It is important to note that the concentrations of dyes in our antibacterial experiments did not exceed 0.023%, which is mainly within the acceptable limits for using food colorants [55]. It is worth mentioning that according to our results, the most active colorant, E127, under illumination killed *S. aureus* at the concentration of 0.004% when combined with 2.2 × 10^−4^% of Rhein (Figure 4b), and it eliminated *E. coli* after short sonication and illumination at the concentration of 0.008% when combined with 4.5 × 10^−4^% of Rhein (Figure 7c). For comparison, according to the Codex General Standard for Food Additives (GSFA, Codex STAN 192-1995), which regulates concentrations of permitted food additives, E127 may be applied in chewing gum, decorations, and candied fruits at 0.005–0.02% [55].

## 3. Materials and Methods

### 3.1. Bacterial Growth

Cultures of *Staphylococcus aureus* (*S. aureus*) (ATCC 25923) and *Escherichia coli* (*E. coli*) (ATCC 10798) were grown on brain–heart agar (BHA; Acumedia, San Bernardino, CA, USA) for 24 h, then transferred into brain–heart infusion broth (BHI; Acumedia, San Bernardino, CA, USA). These were grown at 37 °C for ~2 h with shaking at 170 rpm, reaching a concentration of up to 10^8^ cells/mL (OD_660_ = 0.3). Using a saline solution, the bacterial suspension was diluted by a serial dilution process to reach a final concentration of 10^3^–10^6^ cells/mL.

### 3.2. Preparation of Colorant Stocks

Allura Red AC (E129), Ponceau 4R (E124), Azorubine (E122), Erythrosin B (E127), and Brilliant Blue FCF (E133) were purchased from Glentham Life Sciences Ltd. (Corsham, UK); Rhein was purchased from Angene (Hong Kong, China). The food colorants were dissolved in saline to prepare the following stock solutions: E129 (0.29 mg/mL), E124 (1.40 mg/mL), E122 (0.82 mg/mL), E127 (0.9 mg/mL), and E133 (0.42 mg/mL). E150a was produced by Maimon (Beer Sheva, Israel) as a commercial preparation; it was used as received. Vanillin concentration in E150a (1.82 mg/mL) was determined by calibration curves built using a vanillin standard by spectrophotometric measurements at 315 nm. Rhein was dissolved in 100% ethanol to form the stock solution of 0.05 mg/mL, followed by ultrasound treatment for 30 min in total (three cycles of 10 min in an ultrasonic bath with ice–water replacement) to achieve a homogeneous solution.

### 3.3. Minimal Inhibitory Concentration (MIC) Assay

Using a 24-well ELISA plate, 2.5–2.7 mL of neutral broth (NB) (purchased from Merck, Darmstadt, Germany) and 0.3–0.5 mL of stock colorant solutions were added to the first well, while the rest of the wells in the same line were filled with 1.5 mL of NB. The specific colorant volumes were chosen based on solvent/bacteria type considerations: 0.3–0.5 mL colorant solution in 2.7–2.5 mL NB (in a total 3 mL volume) and applied to Gram-negative bacteria, while 0.3 mL colorant solution in 2.7 mL NB was used for Gram-positive bacteria. Then, the obtained colorant solution was diluted by a serial double dilution procedure, and 100 µL of bacteria at a concentration of 10^6^ cells/mL was added into each well, except the last one, which served as a control for the sterility of the nutrient medium. The well containing only bacteria in NB, without the colorant, served as another control. The plate was exposed to white LED continuous illumination at a distance of 8 cm from the lamp for 30 min while being shaken at 170 rpm using an orbital shaker. Light intensity and fluence rate were 137 klux and 1.6 mW cm^−2^, respectively. Light intensity was measured by an LX-102 light meter (Lutron, Taipei, Taiwan). The plates were incubated for 24 h at 170 rpm in the dark. After incubation, the wells were analyzed visually for turbidity [56,57], and the first transparent well was chosen as the MIC. Here and further in Section 3.6, Section 3.7 and Section 3.8, when testing Rhein against bacteria, the ethanol concentration never exceeded 10%, whereas in the control series, ethanol at this concentration was nontoxic for cells.

### 3.4. Absorption and Fluorescence Measurements

Visible absorption spectra of the colorants were measured with a V-750 UV-visible spectrophotometer (JASCO, Tokyo, Japan) using 10 mm pathlength quartz cuvettes at ambient temperature. The scan rate was 480 nm/min, and the data interval was 1 nm. The emission spectrum of the white LED lamp used for illumination was registered using the HORIBA spectrometer (Jobin Yvon Inc., Edison, NJ, USA) equipped with the monochromator FHR1000 and the detector CCD (HORIBA Scientific’s Synapse™, Irvine, CA, USA). The spectrum was processed by the LabSpec 5 program (HORIBA).

Fluorescence spectra of E127 and Rhein were measured with a spectrofluorometer (Jasco, FP-8350), with the excitation wavelengths for free dyes of 420 nm (Rhein), 500 nm (E127), and 420 nm in FRET (Förster resonance energy transfer) experiments for both dyes and their mixtures.

Relative quantum yields of fluorescence (Ф_F_) were determined using standards of known Ф_F_ [58,59]. Fluorescein (Ф_F_ = 0.92 in 0.1 N NaOH) [60] and coumarin 153 (Ф_F_ = 0.53 in ethanol) [61] were chosen as reference dyes for E127 and Rhein, respectively.

The quantum yields were calculated according to Equation (1) [58,62]:

Ф_F_ = Φ_FRef_ × (F/F_Ref_) × (A_Ref_/A) × (n^2^/n^2^_Ref_), (1)

where Φ_Fref_ is the quantum yield of the reference, F_Ref_ and F are the areas of the emission spectra, A_Ref_ and A are the absorbances at the excitation wavelength, and n^2^ and n^2^_Ref_ are the refractive indices of solvents. The Φ_F_ of E127 was measured in saline and that of Rhein in ethanol.

### 3.5. Singlet Oxygen Production Study

To verify the singlet oxygen generation, the quencher 1,3-diphenyl-isobenzofuran (DPBF) in methanol was used. The absorption of DPBF was monitored at 410 nm at different irradiation or sonication time periods. Rose Bengal (RB) was used as a reference in sonication experiments. The degradation rate of DPBF was determined according to the decrease of the absorption peak at 410 nm versus time.

### 3.6. Photodynamic Cyto-Inactivation Study

Bacterial suspensions with a PS at different concentrations were dispensed in ELISA plates (Falcon^®^ 48-well polystyrene, clear flat bottom) and were incubated together. The incubation of the bacteria with the PS in the dark (pre-irradiation incubation) was carried out for 30 min or 60 min, followed by white LED illumination at a distance of 8 cm from the light source. Control measurements were performed in the dark, with and without a PS. A 100 µL sample from each well was taken and evenly distributed using a Drygalski applicator on Petri-dish agar plates. The plates were incubated for 24 h at 37 °C, the grown colonies were counted using a Scan 500 colony counter (Interscience, Saint-Nom-la-Bretèche, France), and the CFU (colony-forming unit) value was calculated. The number of colonies obtained was multiplied by the dilution factor to calculate surviving cell numbers after the photodynamic treatment.

### 3.7. Ultrasonic Treatment of Cells

The solutions of colorants in different concentrations were dispensed into 1 mL of bacterial suspensions in concentrations of 10^3^–10^4^ cells/mL in 4 mL glass vials and were then mixed. The incubation of the bacteria with a PS in the dark (pre-sonication incubation) was carried out for 30 min, followed by sonication at a frequency of 38 kHz and field strength of 4.1 W cm^−3^, using an ultrasonic bath VUO3H (SMEG Instruments, Guastalla, Italy). In experiments with *E. coli*, the sonication time was 10 or 30 s.

### 3.8. Inactivation of Bacteria by Ultrasonic and Photodynamic Treatment in Series

**Figure 8:**
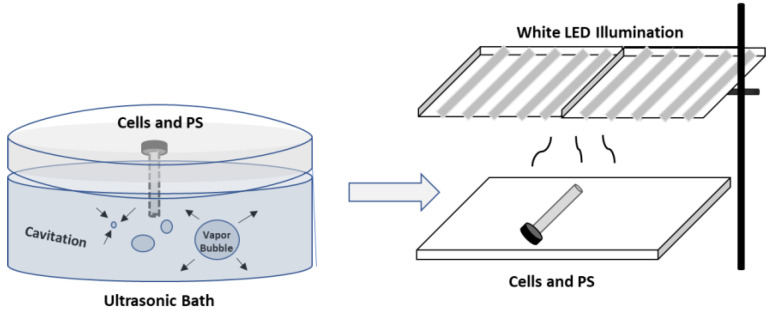
The scheme of sono- and photodynamic bacterial inactivation in series.

Solutions of E127, Rhein, and E127 mixed with Rhein were added to *E. coli* cells, immediately submitted to sonication for 10 s or 30 s, and then exposed to white LED illumination for 5 or 10 min (Figure 8). The experiments were all performed in 4 mL glass vials. Control measurements were performed using dark, light, or ultrasonic treatment only.

### 3.9. Statistics

Each measurement for each PS was repeated three times in duplicate. The difference between the results was considered significant when the *p*-value was less than 0.05. Quantitative results are presented as the mean ± standard deviation (STDEV).

## 4. Conclusions

The photodynamic activity of commonly used edible food colorants was studied against Gram-positive *S. aureus* and Gram-negative *E. coli*. The MIC values depended on the pre-incubation time of colorants with cells. Among the examined colorants, E127 and Rhein demonstrated the highest inhibitory effects against both bacteria.

The illumination of *E. coli* in the presence of E127 or Rhein for 30 min caused a moderate reduction in cell concentration, but the E127/Rhein combination led to a total inactivation of the cells already after 10 min. It is suggested that E127 and Rhein interacted in a supportive mode. The mechanism of their interaction is unclear as yet and may possibly involve FRET interactions, biochemical pathways, or enhanced ROS production.

E127 was found to produce ROS under sonication, whereas Rhein did not exhibit sonodynamic properties. The combined E127/Rhein application led to a profound inactivation of *E. coli* after short sonication followed by illumination. We suggest that a short ultrasonic pre-treatment may replace a long dark pre-incubation.

Using the above-presented sequential sonication and illumination, a combined antibacterial treatment consisting of E127, a synthetic food colorant, and Rhein, a plant derivative, can achieve an effective inactivation of bacteria at reduced photo-sensitizer concentrations and with short treatment times. The result is a potentially safer treatment with reduced side effects and lower energy consumption, while preserving high levels of food quality.

## Figures and Tables

**Table 1:** Chemical structure, photophysical properties, and MIC values of the studied colorants.

Food Colorant	Food Colorant Structure	λ_max_, nm in Visual Region * and Color
E129 Allura Red AC	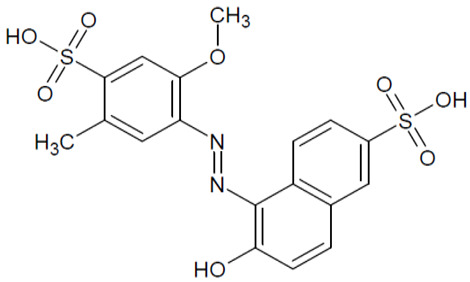	496 Red
E122 Azorubine S.	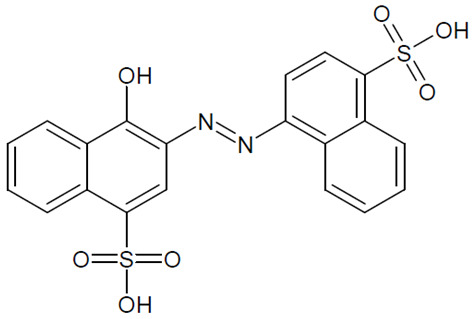	515 Red
E124 Ponceau 4R	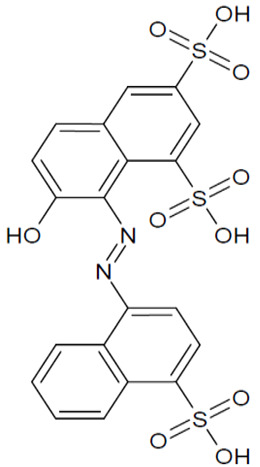	507 Red
E150a Caramel Caramel alkane Caramel olefins Caramel alkynes Vanillin	(C_24_H_36_O_18_)_n_ (C_36_H_50_O_25_)_n_ (C_24_H_36_O_13_)_n_ 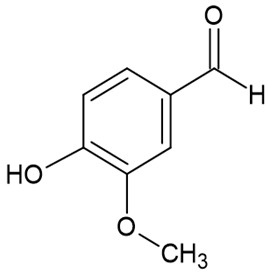	ND ** Reddish brown
E133 Brilliant blue FCF	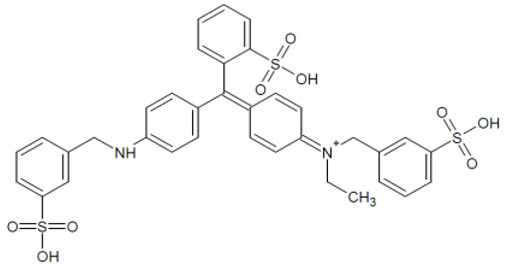	409 and 629 Blue
E127 Erythrosine B	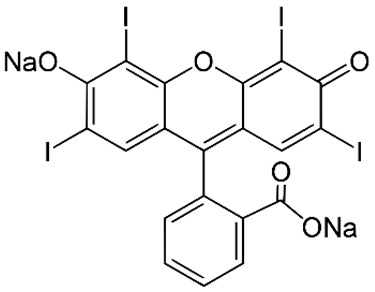	527 Red
Rhein	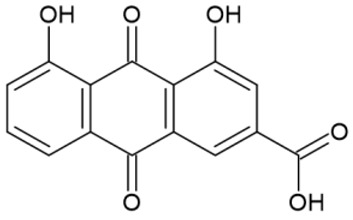	433 Yellow

* Absorption spectra of food colorants were measured in saline, and that of Rhein in ethanol. ** ND—not determined.

## Data Availability

Data are available in this publication.

## Appendix A

Appendix A contains Figure A1 and Figure A2, presented for a better understanding of ROS production measurements in the case of Rhein under illumination and E127 under ultrasonic treatment, respectively.
